# Enablers and Barriers to Deployment of Smartphone-Based Home Vision Monitoring in Clinical Practice Settings

**DOI:** 10.1001/jamaophthalmol.2021.5269

**Published:** 2021-12-16

**Authors:** Edward Korot, Nikolas Pontikos, Faye M. Drawnel, Aljazy Jaber, Dun Jack Fu, Gongyu Zhang, Marco A. Miranda, Bart Liefers, Sophie Glinton, Siegfried K. Wagner, Robbert Struyven, Caroline Kilduff, Darius M. Moshfeghi, Pearse A. Keane, Dawn A. Sim, Peter B. M. Thomas, Konstantinos Balaskas

**Affiliations:** 1 Byers Eye Institute, Stanford University, Palo Alto, California; 2 NIHR Biomedical Research Centre at Moorfields Eye Hospital NHS Foundation Trust, UCL Institute of Ophthalmology, London, United Kingdom; 3 UCL Institute of Ophthalmology, London, United Kingdom; 4 Personalised Healthcare Ophthalmology, F. Hoffmann La Roche AG, Basel, Switzerland; 5 Personalised Healthcare Ophthalmology, Roche Products, Welwyn Gardens City, United Kingdom

## Abstract

**Importance:**

Telemedicine is accelerating the remote detection and monitoring of medical conditions, such as vision-threatening diseases. Meaningful deployment of smartphone apps for home vision monitoring should consider the barriers to patient uptake and engagement and address issues around digital exclusion in vulnerable patient populations.

**Objective:**

To quantify the associations between patient characteristics and clinical measures with vision monitoring app uptake and engagement.

**Design, Setting, and Participants:**

In this cohort and survey study, consecutive adult patients attending Moorfields Eye Hospital receiving intravitreal injections for retinal disease between May 2020 and February 2021 were included.

**Exposures:**

Patients were offered the Home Vision Monitor (HVM) smartphone app to self-test their vision. A patient survey was conducted to capture their experience. App data, demographic characteristics, survey results, and clinical data from the electronic health record were analyzed via regression and machine learning.

**Main Outcomes and Measures:**

Associations of patient uptake, compliance, and use rate measured in odds ratios (ORs).

**Results:**

Of 417 included patients, 236 (56.6%) were female, and the mean (SD) age was 72.8 (12.8) years. A total of 258 patients (61.9%) were active users. Uptake was negatively associated with age (OR, 0.98; 95% CI, 0.97-0.998; *P* = .02) and positively associated with both visual acuity in the better-seeing eye (OR, 1.02; 95% CI, 1.00-1.03; *P* = .01) and baseline number of intravitreal injections (OR, 1.01; 95% CI, 1.00-1.02; *P* = .02). Of 258 active patients, 166 (64.3%) fulfilled the definition of compliance. Compliance was associated with patients diagnosed with neovascular age-related macular degeneration (OR, 1.94; 95% CI, 1.07-3.53; *P* = .002), White British ethnicity (OR, 1.69; 95% CI, 0.96-3.01; *P* = .02), and visual acuity in the better-seeing eye at baseline (OR, 1.02; 95% CI, 1.01-1.04; *P* = .04). Use rate was higher with increasing levels of comfort with use of modern technologies (β = 0.031; 95% CI, 0.007-0.055; *P* = .02). A total of 119 patients (98.4%) found the app either easy or very easy to use, while 96 (82.1%) experienced increased reassurance from using the app.

**Conclusions and Relevance:**

This evaluation of home vision monitoring for patients with common vision-threatening disease within a clinical practice setting revealed demographic, clinical, and patient-related factors associated with patient uptake and engagement. These insights inform targeted interventions to address risks of digital exclusion with smartphone-based medical devices.

## Key Points

**Question:**

What are the enablers and barriers of patient engagement for app-based home vision monitoring at scale?

**Findings:**

In this cohort and survey study including 417 adults, 258 patients were active users (61.9%) of whom 166 patients (64.3%) were compliant users. Engagement was positively associated with higher comfort with technology, White British ethnicity, visual acuity, neovascular age-related macular degeneration diagnosis, and the number of intravitreal injections and was negatively associated with increased age.

**Meaning:**

These findings suggest effective smartphone app-based home vision monitoring should address the risk factors for low engagement and digital exclusion during clinical practice setting deployment.

## Teaser

This cohort study examines the associations between patient characteristics and clinical measures with vision monitoring app uptake and engagement.

## Introduction

A patient-centric approach to modern medicine involves bringing care not only to patients’ homes but also to the palm of their hands. Recent social distancing practices along with advancements in digital technologies have shone a light on the potential for telemedicine in medicine and ophthalmology.^1,2^ New care models are emerging to monitor chronic diseases through home data collection, reducing unnecessary hospital visits. This is especially beneficial for patients with difficulty traveling to regular appointments, such as older adults and those with blinding eye conditions.

Home health monitoring has been used for chronic diseases, such as hypertension, dementia, chronic obstructive pulmonary disease, heart failure, and glaucoma, and has been shown to reduce cost while improving outcomes.^3,4,5,6,7,8^ Ophthalmology hospital attendances account for 10% for all outpatient activity in the United Kingdom, more than any other individual medical specialty. Modern ophthalmic practice is faced by the challenges of an aging population, increasing disease prevalence, and the need for regular monitoring of chronic conditions, such as age-related macular degeneration (AMD) and diabetic retinopathy. This leads to an immense strain on diagnostic services, reducing timely access to care.

AMD has leveraged a rudimentary method for remote vision monitoring, the Amsler grid, which consists of self-reported distortion on a grid of intersecting lines.^9^ Digital technologies, such as home vision monitoring devices and smartphone-based apps, have recently enabled reliable monitoring of visual function for patients with common retinal disease, allowing earlier disease detection and treatment.^10,11,12,13^

In contrast to passive monitoring, solutions that encourage active patient engagement may lead to improved health outcomes.^14,15^ It is reasonable to infer that an app that increases patient engagement through reminders may lead to improved compliance and clinical outcomes. Potential applications of home vision monitoring include early detection of progression from intermediate to neovascular AMD (nAMD), fellow-eye conversion in patients with nAMD in the first eye, as well as monitoring patients receiving anti–vascular endothelial growth factor (VEGF) treatment for nAMD, diabetic macular edema or macular edema secondary to retinal vein occlusions. An important consideration for real-world deployment of home vision monitoring at scale is assessing potential enablers and barriers to patient uptake and compliance. Strategies may then be developed to address digital exclusion, particularly in vulnerable patient populations, such as older adults and those with visual impairment.

In this work, we report outcomes of a clinical practice setting (real-world) deployment of a smartphone-based home vision monitoring app, Home Vision Monitor (HVM; also known as myVisionTrack [mVT; Vital Art and Science]), in patients attending a high-volume intravitreal injection clinic. To assess enablers and barriers to uptake and compliant use, we conducted a comprehensive assessment of demographic, clinical, and perception-related factors.

## Methods

### Setting and Patient Population

Consecutive patients attending Moorfields Eye Hospital (MEH) between May 2020 and February 2021 for planned anti-VEGF intravitreal injections who possessed a smartphone or tablet between May 2020 and February 2021 were offered the HVM smartphone app and advised to self-test twice weekly. Process for patient approach and registration is described in the eMethods in the Supplement. This app was linked with a web-based data store and associated physician review portal. The HVM app has received US Food and Drug Administration 510k clearance and CE marking and uses shape discrimination hyperacuity to detect metamorphopsia in the central degrees of vision as a metric of visual function.^16,17^ A change in visual function equivalent to a predefined threshold of 2 SDs above the baseline on the logMAR scale on 2 consecutive occasions triggered an alert message to the clinician. The patient was contacted, and early recall to the clinic was initiated based on symptom triage.

HVM was introduced as a service quality improvement initiative at MEH in May 2020. The project received information governance approval and clinical safety approval. The collection and analysis of data on HVM performance, including a patient engagement survey, was conducted through a clinical audit registered and approved by the MEH Clinical Audit department. The requirement for individual patient consent for data analysis and reporting is waived for the purposes of National Health System clinical practice auditing.

The demographic variables included were age, biological sex, and ethnicity. Ethnicity was obtained from the patient’s hospital record, as one of 12 self-reported categories, including Black African, Black Caribbean, Indian, Pakistani, Bangladeshi, White British, Irish, and other Black, other Asian, or other White backgrounds. Unknown ethnicity or not stated by participants were also possible categories. Biological sex was self-reported as either male or female. Age was defined as the patient’s age at baseline.

### Metrics of Patient Engagement

Uptake was defined as successful installation and subsequent use of the app at least once. Patients who met this requirement were defined as active users. Engagement for active app users was assessed via compliance and use rate. Compliance was defined as any continuous period of at least 4 weeks during which vision tests were performed at least twice weekly. Mean use rate represents the total number of tests conducted by a patient divided by the overall period (in weeks) since the app was prescribed. Types and definitions of demographic and clinical predictive variables included in the analysis as well as content development and delivery of the patient survey are described in the eMethods in the Supplement.

### Statistical Analysis

Associations between engagement metrics (uptake, compliance, app use rate) and predictor variables were analyzed. Univariable analysis was performed for demographic and clinical variables for all participants. Univariable analysis for the 18 categorical or ordinal survey variables was performed for participants that had completed the survey (eTable 2 in the Supplement). Multivariable analysis of observed associations was performed using logistic regression for a binary outcome or linear regression for a continuous outcome. Select survey questions were included in a machine learning random forest analysis to provide a feature importance ranking, indicating the relative weight of the corresponding variable influencing patient engagement. Descriptive statistics were performed as summarized in the eMethods in the Supplement.

eTable 2 in the Supplement summarizes the types of statistical tests that were performed depending on whether the predictor variable was categorical, ordinal, or continuous and whether the outcome variable was binary or continuous. Significance was set at a *P* value less than .05, and all *P* values were 2-tailed. All statistical analysis was conducted using R version 3.5.1 (The R Foundation).

## Results

**Figure 1.**
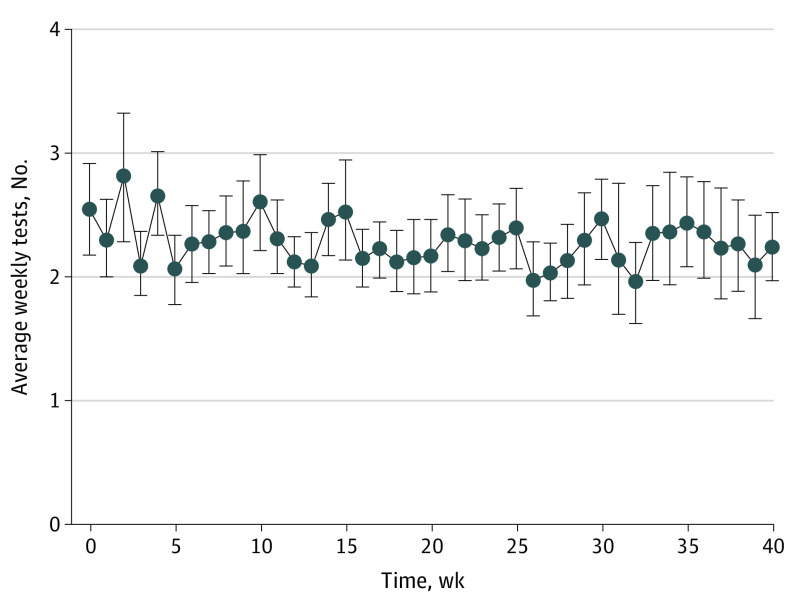
Mean App Usage by Study Week in Active Patients Each point represents the mean number of weekly app-based vision tests per patient. Only positive usage values are included; zero usage at each week was omitted. Error bars represent 95% CIs.

Of 417 included patients, 236 (56.6%) were female, and the mean (SD) age was 72.8 (12.8) years. A total of 258 patients (61.9%) were active users and 159 (38.1%) were nonactive. Of 258 active users, 166 patients (64.3%) fulfilled the definition of compliance, with 92 (35.7%) classified as noncompliant (eFigure 1 in the Supplement). Mean (SD) weekly app use (at week 4) was 1.83 (2.46). Mean weekly use rate over time is presented in Figure 1.

### Demographic and Patient Characteristics

Patient cohort characteristics are summarized in Table 1. A total of 289 patients (69.9%) had a diagnosis of nAMD, 109 (26.3%) had a diagnosis of macular retinal edema, and 15 (3.7%) had another diagnosis. The median (range) of visual acuity (VA) at baseline was 80 (23-97) Early Treatment Diabetic Retinopathy Study (ETDRS) letters in the better-seeing eye and 63 (0-95) ETDRS letters in the worse-seeing eye. Patients received a median (range) of 18 (0-154) injections at baseline. Distributions of VA and injection numbers are presented in eFigure 2 in the Supplement. Female patients were older than male patients (eFigure 3 in the Supplement). Mean HVM scores and VA values for right and left eyes over time are presented in eFigure 4 in the Supplement.

**Table 1.** Demographic and Clinical Properties of All 417 Participants

**Variable**	**No. (%)** ^a^	
	**Male (n = 177)**	**Female (n = 236)**
**Demographic** ^b^		
Age, mean (SD), y	71.5 (13.6)	73.8 (12.0)
Ethnicity		
Asian or Asian British	18 (10.2)	21 (8.9)
Black or Black British	4 (2.3)	4 (1.7)
White British	83 (46.9)	137 (58.1)
Any other White background	19 (10.7)	32 (13.6)
Not stated	50 (28.3)	36 (15.3)
Unknown	3 (1.7)	6 (2.5)
**Clinical**		
Diagnosis		
AMD	119 (67.2)	170 (72.0)
MRO	55 (31.1)	54 (22.9)
Not available	3 (1.7)	12 (5.1)
Visual acuity, mean (SD), ETDRS letters		
Better-seeing eye	76.3 (13.3)	75.4 (12.8)
Worse-seeing eye	58.5 (21.9)	57.5 (21.1)
Active users^c^		
Left eye		
Baseline	73.2 (15.2)	68.5 (12.6)
At week 17	57.8 (22.3)	72.8 (10.9)
At week 35	73.0 (16.2)	72.3 (16.2)
Right eye		
Baseline	66.0 (22.4)	57.7 (23.6)
At week 17	62.7 (25.6)	69.1 (19.2)
At week 35	73.2 (17.4)	71.8 (17.5)
All users		
Left eye		
Baseline	73.3 (15.2)	68.5 (68.5)
At week 17	63.9 (22.2)	68.4 (17.4)
At week 35	73.6 (15.0)	71.8 (16.0)
Right eye		
Baseline	66.0 (22.3)	57.7 (61.7)
At week 17	65.3 (21.7)	69.0 (16.8)
At week 35	73.0 (16.7)	70.7 (17.5)
Injections, mean (SD), No.	26.6 (26.9)	30.5 (29.2)
**HVM-app**		
SDH score, mean (SD)		
Left eye		
Baseline	−0.166 (0.452)	−0.138 (0.447)
At week 17	−0.290 (0.389)	−0.249 (0.353)
At week 35	−0.002 (0.441)	0.022 (0.448)
Right eye		
Baseline	−0.168 (0.450)	−0.130 (0.450)
At week 17	−0.303 (0.371)	−0.228 (0.353)
At week 35	0.015 (0.475)	0.027 (0.435)
Mean uses per week, mean (SD)		
At week 4	1.500 (2.416)	2.045 (2.483)
At week 17	1.069 (1.475)	1.116 (2.136)
At week 35	0.196 (1.437)	0.123 (1.092)
Outcome variables		
Active	102 (57.6)	155 (65.7)
Compliant	63 (35.6)	103 (43.6)
Engagement rate	0.33 (0.17)	0.34 (0.16)
Survey completed	48 (27.1)	73 (30.9)

Abbreviations: AMD, age-related macular degeneration; ETDRS, Early Treatment Diabetic Retinopathy Study; HVM, Home Vision Monitor; MRO, macular retinal edema; SDH, shape discrimination hyperacuity.

^a^ A total of 4 participants (1 active user and 3 nonactive users) did not have a retrievable electronic health record.

^b^ Demographic data, including age, biological sex, and self-reported ethnicity, as well as clinical data were obtained from the patients’ electronic health record and were thus available for all patients.

^c^ Within all participants, 258 of those were active users.

### Associations With Patient Engagement

#### Uptake

VA in the better-seeing eye and number of injections at baseline were positively associated with uptake (eTable 3 in the Supplement). Each ETDRS line of better VA in the better-seeing eye was associated with a 1.7% increase in likelihood of being an active user (OR, 1.02; 95% CI, 1.00-1.03; *P* = .01). Every additional injection received before baseline was associated with a 0.8% increase in likelihood of being an active user (OR, 1.01; 95% CI, 1.00-1.02; *P* = .02). Increasing age was negatively associated with uptake. An increase of 1 year of age led to a 1.8% decrease in likelihood of being an active user (OR, 0.98; 95% CI, 0.97-0.998; *P* = .02).

#### Compliance

**Figure 2.**
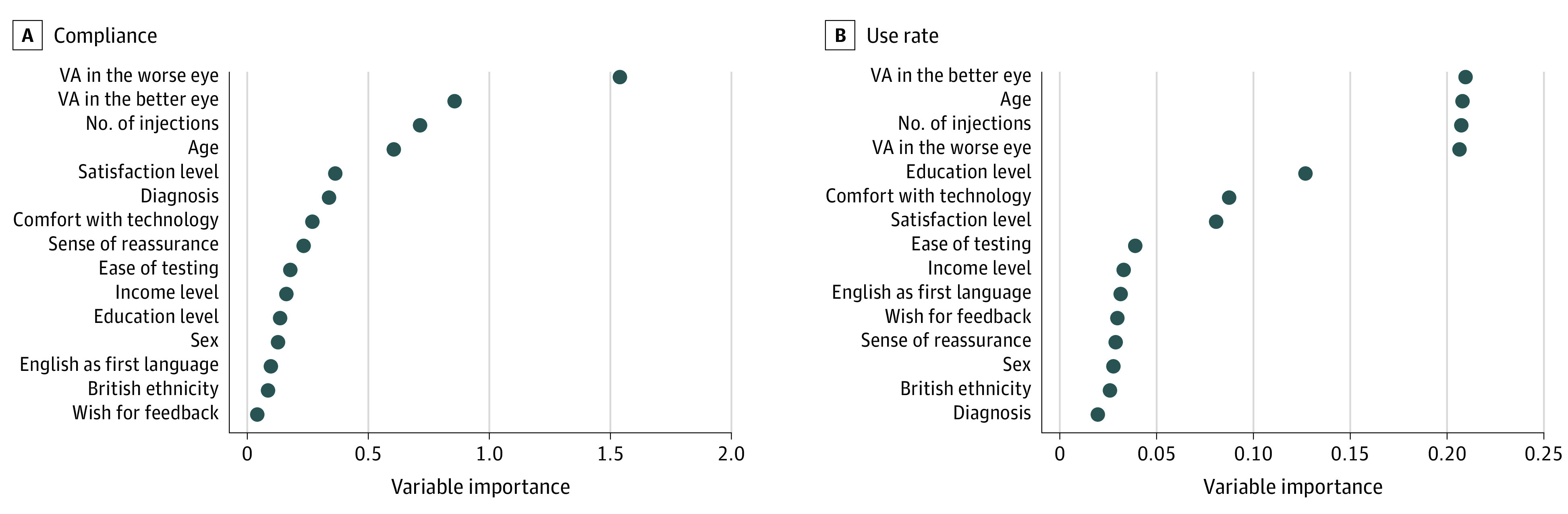
Machine Learning Feature Importance Analysis (Random Forest) Feature importance of variables is provided for compliance (binary outcome; compliant vs noncompliant; loss function: gini impurity) and use rate (linear outcome; range, 0 to 1; loss function: mean squared error). VA indicates visual acuity.

Clinical diagnosis, VA in the better-seeing eye, and White British ethnicity were associated with compliance (Table 2). However, the association with White British ethnicity was not maintained after controlling for education, income level, and comfort with use of modern technologies, likely reflecting ethnic population-level disparities. Patients with nAMD were more likely to be compliant (131 of 289 [45.3%]) than patients with macular retinal edema (32 of 109 [29.3%]; OR, 1.94; 95% CI, 1.07-3.51; *P* = .002). Every line of improved VA on the ETDRS chart in the better-seeing eye at baseline had a 2.1% increase in likelihood of being a compliant user (OR, 1.02; 95% CI, 1.01-1.04; *P* = .04). In the group that completed the patient experience survey, compliance had a negative association with the wish of the patient to receive test results (OR, 0.82; 95% CI, 0.71-0.95; *P* = .01). This association persisted after adjusting for ethnicity, diagnosis, and VA in the better-seeing eye. Feature importance analysis (machine learning) demonstrated high relative importance of age, number of prior injections, and baseline vision. Educational level, comfort with modern technologies, and satisfaction with app use demonstrated moderate feature importance (Figure 2).

**Table 2.** Association of Patient Compliance With Demographic, Clinical, and Survey Predictor Variables

Variable	No. (%)		Univariable analysis		Multivariable analysis	
	Compliant	Not compliant	OR (95% CI)	*P* value	OR (95% CI)	*P* value
Age						
Median (IQR), y	75.0 (67.0-80.0)	75.0 (67.0-83.0)	1.02 (0.99-1.03)	.28	NA	NA
Missing	0	4 (1.6)				
Biological self-reported sex						
Female (baseline)	103 (62.0)	133 (53.0)	0.71 (0.47-1.09)	.07	NA	NA
Male	63 (38.0)	114 (45.4)				
Ethnicity						
British	81 (48.8)	94 (37.5)	1.69 (0.96-3.01)	.02	1.43 (0.43-4.90)	.56
Not British (baseline)	85 (51.2)	153 (61.0)				
Diagnosis						
AMD	131 (78.9)	158 (63.7)	1.94 (1.07-3.53)	.002	2.48 (0.68-7 8.63)	.16
MRO (baseline)	32 (19.3)	77 (30.7)				
VA in worse eye						
Median (IQR), ETDRS letters	64.0 (45.0-73.0)	63.0 (45.0-75.0)	1.00 (0.99-1.01)	.95	NA	NA
Missing	2 (1.2)	6 (2.4)				
VA in better eye						
Median (IQR), ETDRS letters	80.0 (72.0-85.0)	78.0 (70.0-84.0)	1.02 (1.01-1.04)	.04	1.03 (0.98-1.08)	.13
Missing	2 (1.2)	6 (2.4)				
Injections						
Median (IQR), No.	20.0 (9.2-45.0)	17.0 (7.0-41.5)	1.06 (1.02-1.10)	.08	NA	NA
Missing	0	4 (1.6)				
Wish to receive results						
Yes	68 (68.0)	16 (94.1)	0.82 (0.71-0.95)	.01	0.87 (0.75-1.02)	.02
No	29 (29.0)	0				
Missing	3 (3.0)	1 (5.9)				

Abbreviations: AMD, age-related macular degeneration; ETDRS, Early Treatment Diabetic Retinopathy Study; MRO, macular retinal edema; OR, odds ratio; VA, visual acuity.

#### Weekly Mean Use Per Patient

Lower vision in the worse-seeing eye (β = 0.001; 95% CI, 0-0.002; *P* = .03) and higher number of previous injections (β = 0.001; 95% CI, 0-0.001; *P* = .03) were associated with an increased weekly use rate (eTable 4 and eFigure 5 in the Supplement). On the 5-point scale used to measure comfort with technology, every additional point was associated with a 2.4% increase in use rate (β = 0.024; 95% CI, 0-0.047; *P* = .05). This association was maintained after adjusting for both VA in the worse-seeing eye and number of injections (β = 0.031; 95% CI, 0.007-0.055; *P* = .02).

### Patient Survey

**Figure 3.**
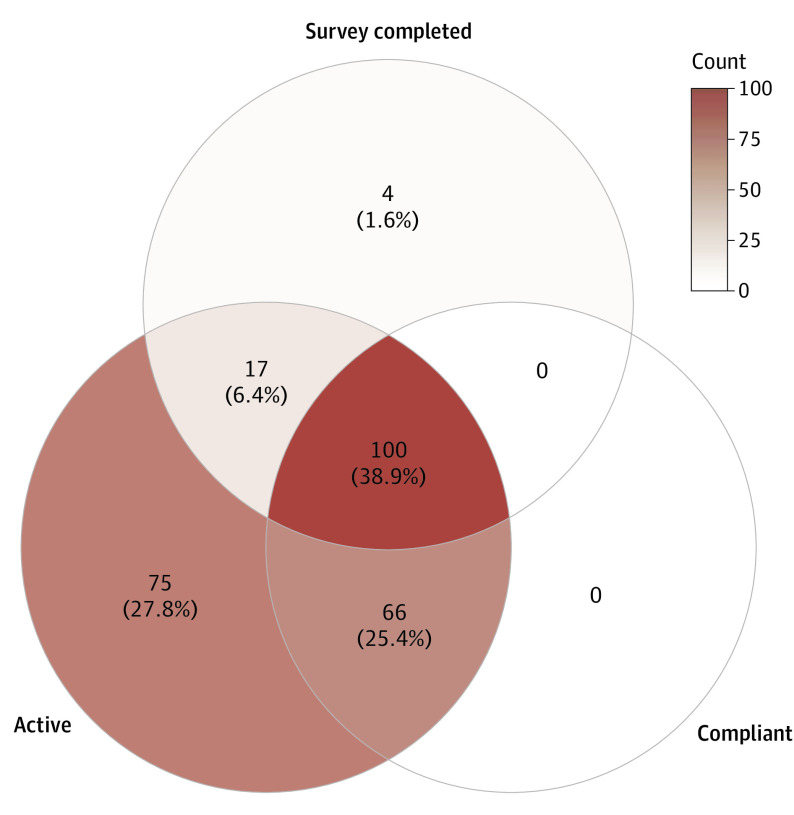
Patient Uptake, Compliance, and Survey Completion Rates Number and percentage of overlapping patients within each circle, shaded by count.

Of 117 active users completing the survey (Figure 3), 100 (85.5%) were compliant. The correlation matrix for answered questions is presented in eFigure 6 in the Supplement. The aggregate survey answer distributions are presented in eTable 1 in the Supplement. Statistical associations between survey questions and compliance are presented in eTables 5 and 6 in the Supplement.

### Patient-Specific Characteristics

Level of comfort with modern technologies had a strong association with higher levels of app use rate, even controlling for age, diagnosis, and VA (β = 0.031; 95% CI, 0.007-0.055; *P* = .02). A total of 78 patients (69.9%) reported they were either comfortable or very comfortable with use of modern technologies. A total of 88 patients (75.2%) reported English as their first language, which was not associated with a difference in compliance or use rate. Five patients expressed desire for the instructions to be available in different languages.

### Patient Motivation and Satisfaction

The most common motivation to use the HVM app (65 of 117 [55.6%]) was the expectation that closer vision monitoring had the potential to benefit patients’ eye health. Patient approach and registration process to the HVM app are summarized in eFigure 7 in the Supplement. App users reported high levels of satisfaction, with 108 patients (92.3%) giving a rating of 3 or higher (score range, 1 to 5), 94 (80.3%) reported the app met expectations (a score of 3 or higher), and 101 (88.5%) would recommend the app to others.

### Ease of Use

A total of 119 patients (98.3%) found the app either easy or very easy to use, 93 of 112 (83.0%) required no further assistance for testing their vision, and 96 of 117 (82.1%) experienced a sense of increased reassurance from the knowledge that using the HVM app would allow clinicians to more closely monitor their vision. A total of 34 patients (28.8%) required assistance downloading and installing the app.

### Patient Feedback for Improvement

Patients expressed an overwhelming wish (103 of 117 [88.0%]) to receive feedback about their vision test results at test time. A total of 53 patients (45.3%) would wish to view their vision test results every time, while 64 (54.7%) would wish to receive feedback only when substantial changes in vision would be detected. A total of 97 responses (63.4%) to a choose-all-that-apply question with recommendations for improvements by patients highlighted more feedback about their vision test results as a key motivating factor for continuous engagement.

### Vision Change Alerts

A total of 26 alerts for substantial vision worsening were triggered; 18 of these patients also subjectively noticed a change in vision, while 8 did not. A total of 11 patients had their treatment appointment moved sooner, and the remainder had preplanned appointments in the retinal treatment clinic within 7 days of the alert. In 22 of 26 alerted patients, active disease was detected during the clinic visit, and patients were subsequently treated with an anti-VEGF injection (sensitivity, 84.6%). A total of 3 patients presented with stable disease as false-positives (specificity, 88.5%). One patient had a retinal detachment, which was treated.

## Discussion

For home health monitoring to acquire a substantial role in health care systems, it needs to have meaningful uptake and continued patient engagement. We must consider patient-specific barriers to scaling these technologies, as they may lead to digital exclusion. This is particularly relevant for home vision monitoring, as it involves a vulnerable, older, and visually impaired patient population.

Home vision monitoring with portable devices and smartphone-based apps has shown potential for common medical retinal disease.^13,18,19^ The Notal Vision Foresee Home was shown to pick up more patients converting from dry AMD to nAMD than office visits.^20^ Shape discrimination hyperacuity–based apps have also been used to detect fellow-eye conversion to nAMD.^11^ Similar benefits for the management of patients with nAMD and diabetic macular edema have been demonstrated from the use of smartphone-based home vision monitoring apps in controlled research environments.^21^ For real-world translation of such benefits to occur, potential barriers to adoption not manifested in controlled research environments require careful consideration. We report on an extended deployment of HVM in a high-volume clinical setting. By using a combination of electronic health records, HVM app metrics, and patient surveys, we performed an in-depth analysis of variables for patient uptake, engagement, and comfort.

### Uptake and Engagement

The strongest predictor of uptake in our patient cohort was age, with older individuals relatively less likely to become active users, representing a risk factor for digital exclusion. A positive association with both uptake and compliance was found with better vision in the better-seeing eye. While a causative relationship cannot be assumed, better vision may be associated with smartphone familiarity in a given patient’s past or a strong motivation to preserve existing vision. Compliance was higher for patients with nAMD than macular retinal edema, even after accounting for age, VA, and ethnicity. This correlates with previously reported higher attendance and compliance for patients with nAMD compared with those with diabetic retinopathy.^22,23^ Although 24.8% of surveyed patients reported a first language other than English, this did not have an association with uptake or engagement. Machine learning models that generate feature importance rankings may increase interpretability of observed associations. Our model’s rankings (random forest) largely confirmed the characteristics from our regression analysis while also highlighting education level and app satisfaction as having an impact on compliance and use rate.

Our observed high levels of compliance for patients with nAMD are encouraging for real-world use of home vision monitoring. However, this does not negate the need to develop effective strategies against digital exclusion for those with worse vision and diabetes and those of older age. A strong association was found between weekly use rate and surveyed comfort with modern technologies, suggesting substantial potential gains by increasing digital literacy through educational initiatives, patient outreach, feedback, and support networks.

### Patient Feedback

Qualitative elements of patient feedback were captured through open-ended survey questions. A common theme for those with a positive experience of the app was a sense of not being forgotten and reassurance that any worsening in their eye health will be notified to the clinical team. Feedback from patients with poor experience from app use highlighted a sense of apprehension about their ability to perform the test correctly and occasional frustration with the repetitive nature of the task.

Most patients expressed a strong preference toward feedback of their results, which the HVM app currently does not provide. This is intended to prevent concern from intersession fluctuations in visual function. Patients who desired more frequent feedback on their test results were more likely to disengage from app use, demonstrating a strong negative association with compliance.

### App Performance Metrics

Notification alerts to the clinical team for vision changes demonstrated high sensitivity (84.6%) and specificity (88.5%). This portends clinical utility and better performance than the widely used Amsler grid.^9^

### Future Directions

Simply initiating smartphone-based home monitoring is insufficient for clinical impact. An integrated cloud-based platform is preferred, to enable ingestion of structured data from these technologies into the clinic.^24,25^ Open standards, such as Fast Healthcare Interoperability Resources and Health Level 7, are required and being developed for home monitoring.^26^

Although potential benefits for home monitoring have been elucidated, less is known about its perception by older populations. Elucidative focus groups by Wild et al^27^ demonstrated 4 dominant themes of patient desires: maintaining independence, detecting cognitive decline, sharing of information, and the tradeoff between privacy and usefulness of monitoring. Beyond patient perceptions, effective and efficient home monitoring faces further hurdles, including incompatible information technology systems, lack of evaluation frameworks for legal, ethical, and technical aspects, and lack of guidelines for implementation.^28^ Our study helps frame these issues for future investigation, which are active research areas for our group.

### Limitations

Our study has limitations. The definition of appropriate metrics to describe human behavior, such as uptake and engagement, is inherently challenging, and their applicability in other practice settings has not been evaluated. HVM app uptake failure represents willing patients unable to use the app but does not capture the unwilling patients. The survey queried active users and is only informative for compliance of those users. The survey cohort had an overall self-reported high level of education and comfort with technologies. We must be cautious in generalizing our results to those with lower education levels or backgrounds differing from our cohort’s distribution. Additionally, our deployment included proactive measures to maintain patient engagement through follow-up calls and reminders. The extent of proactive patient support will likely vary in other settings.

## Conclusions

Patient engagement is crucial for meaningful real-world use of home health monitoring. In this work, we examined enablers and barriers of engagement with app-based home vision monitoring of retinal conditions. We combined app-generated data and electronic health record data along with surveys to discern a number of demographic, clinical, and patient factors affecting uptake and compliance. As health care systems increasingly adopt digital and remote models of care, issues such as digital exclusion require careful consideration to both ensure equitable access and avert disparate outcomes.

## Notes

Supplement.
eMethods.
**eFigure 1.** Types of users of the Home Vision Monitoring app and outcome variables. **eFigure 2.** Distribution of visual acuity in better- and worse-seeing eye (ETDRS letter score) and number of injections at baseline. **eFigure 3.** Age distribution of participants (a) and age distribution between female and male participants (b). **eFigure 4.** Change in SDH score and visual acuity. **eFigure 5.** Histogram of use rate. **eFigure 6.** Correlation matrix of patient survey responses (Pearson correlation coefficients). **eFigure 7.** Patient registration flow chart—Home Vision Monitoring. **eTable 1.** Survey questions and responses. **eTable 2.** Type of univariable statistical tests performed depending on type of predictor and outcome variable. **eTable 3.** Association of patient uptake with demographic and clinical predictor variables (active vs nonactive users). **eTable 4.** Associations of patient engagement rate with demographic, clinical, and survey predictor variables. **eTable 5.** Univariable associations between patient compliance outcome and before-usage survey variables (compliant vs noncompliant users). **eTable 6.** Univariable associations between patient compliance outcome and after-usage survey variables (compliant vs noncompliant users).
