# Supplementary material for: Enablers and Barriers to Deployment of Smartphone-Based Home Vision Monitoring in Clinical Practice Settings
Source: JAMA Ophthalmol. 2021 Dec 16;140(2):153–60. doi: 10.1001/jamaophthalmol.2021.5269 (PMC8678899; doi:10.1001/jamaophthalmol.2021.5269)
Supplement: Supplement. — eMethods. eFigure 1. Types of users of the Home Vision Monitoring app and outcome variables. eFigure 2. Distribution of visual acuity in better- and worse-seeing eye (ETDRS letter score) and number of injections at baseline. eFigure 3. Age distribution of participants (a) and age distribution between female and male participants (b). eFigure 4. Change in SDH score and visual acuity. eFigure 5. Histogram of use rate. eFigure 6. Correlation matrix of patient survey responses (Pearson correlation coefficients). eFigure 7. Patient registration flow chart—Home Vision Monitoring. eTable 1. Survey questions and responses. eTable 2. Type of univariable statistical tests performed depending on type of predictor and outcome variable. eTable 3. Association of patient uptake with demographic and clinical predictor variables (active vs nonactive users). eTable 4. Associations of patient engagement rate with demographic, clinical, and survey predictor variables. eTable 5. Univariable associations between patient compliance outcome and before-usage survey variables (compliant vs noncompliant users). eTable 6. Univariable associations between patient compliance outcome and after-usage survey variables (compliant vs noncompliant users). [file jamaophthalmol-e215269-s001.pdf]

Supplemental Online Content

Korot E, Pontikos N, Drawnel FM, et al. Enablers and barriers to deployment of smartphone-based home vision monitoring in clinical practice settings. *JAMA Ophthalmol*. Published online December 16, 2021. doi:10.1001/jamaophthalmol.2021.5269

eMethods.
eFigure 1. Types of users of the Home Vision Monitoring app and outcome variables
eFigure 2. Distribution of visual acuity in better- and worse-seeing eye (ETDRS letter score) and number of injections at baseline
eFigure 3. Age distribution of participants (a) and age distribution between female and male participants (b)
eFigure 4. Change in SDH score and visual acuity
eFigure 5. Histogram of use rate
eFigure 6. Correlation matrix of patient survey responses (Pearson correlation coefficients)
eFigure 7. Patient registration flow chart—Home Vision Monitoring
eTable 1. Survey questions and responses
eTable 2. Type of univariable statistical tests performed depending on type of predictor and outcome variable
eTable 3. Association of patient uptake with demographic and clinical predictor variables (active vs nonactive users)
eTable 4. Associations of patient engagement rate with demographic, clinical, and survey predictor variables
eTable 5. Univariable associations between patient compliance outcome and before-usage survey variables (compliant vs noncompliant users)
eTable 6. Univariable associations between patient compliance outcome and after-usage survey variables (compliant vs noncompliant users)

This supplementary material has been provided by the authors to give readers additional information about their work.

© 2021 Korot E et al. *JAMA Ophthalmology*.

eMethods.

Process for Patient Approach and Registration

Patients who agreed to use the application were offered informational material and assistance to download the app either at their appointment, or at home at their convenience. Patients were followed up at 2 weeks, 1 month and every 3 months either in the clinic or by telephone. During the follow-up calls, non-compliant patients were reminded to use the application twice a week and were also encouraged to activate the reminder feature in the app settings.

Definitions of Dependent and Independent Variables

Predictive Variables

All time-dependent variables were defined at baseline. Baseline was defined as either the start of the project (May 2020) or first-use of the app (May 2020 - January 2021).

Demographic Variables

Ethnicity was defined according to the patient's hospital record into one of twelve self-reported ethnic groups. Given that some categories had a small sample size (less than 5), the ethnicity variable was also dichotomized into a white-British / non white-British variable. Biological sex was self-reported as either male or female. Age was defined as the patient's age at baseline.

Clinical Variables

Clinical variables included distance visual acuity with correction (in the ETDRS scale), total number of previous anti-VEGF injections at baseline, and clinical diagnosis. Distance visual acuity was defined at baseline and grouped as visual acuity in the worse seeing eye and visual acuity in the better seeing eye. The diagnosis was defined as either nAMD, macular

© 2021 Korot E et al. *JAMA Ophthalmology*.

retinal edema (MRE) or other. The MRE diagnosis grouped together cases of macular edema (ME) associated with DME and retinal vein occlusions (RVOs).

Patient Survey Content Development and Delivery

A patient-centered survey was iteratively developed with early feedback from participating patients, obtained at the time of patient on-boarding and registration during the first month of implementation of the home-vision monitoring service quality improvement initiative. The survey was conducted either in person or over the phone. In each case, patients were approached for completing the survey at 2 months from commencement of app use for each individual patient.

The survey included 26 questions (Supplementary Table 1), of which 18 were close-ended and generated structured pre-selected answers which were transformed into either categorical or ordinal variables for the purposes of the analysis. The questions were grouped as general (16 questions), experience with HVM app set-up (4 questions), experience with vision testing (4 questions), app features and improvement (5 questions) and reasons for continued engagement (1 question). 7 open-ended questions generated free text answers.

Outcome Analysis

Descriptive statistics for patient demographic, clinical, socioeconomic, cultural and perception-related characteristics were performed, and aggregate metrics of visual function (HVM scores and VA values from the EHR) were derived. Patient experience and satisfaction with home vision monitoring, ease of use, frequency of use and its fluctuation over time were reported. An analysis of trigger events for significant vision change in terms of frequency, false positive and false negative rates was also performed. Data from the HVM portal, the EHR, and the patient surveys were used to inform an analysis of parameters influencing patient, compliance and app use rate.

© 2021 Korot E et al. *JAMA Ophthalmology*.

eFigure 1. Types of users of the Home Vision Monitoring app and outcome variables

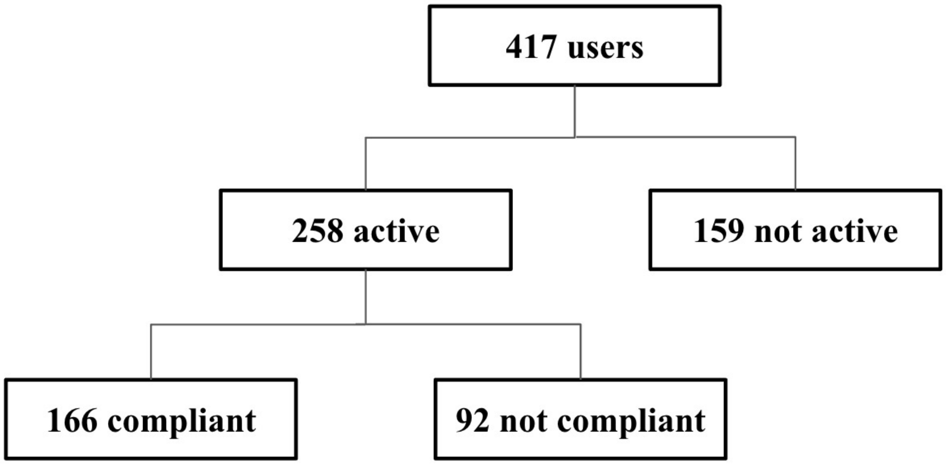

© 2021 Korot E et al. *JAMA Ophthalmology*.

eFigure 2. Distribution of visual acuity in better- and worse-seeing eye (ETDRS letter score) and number of injections at baseline

(a) Distribution of number of injections at baseline (b) Distribution of visual acuity in the worse eye (c) Distribution of visual acuity in the better eye

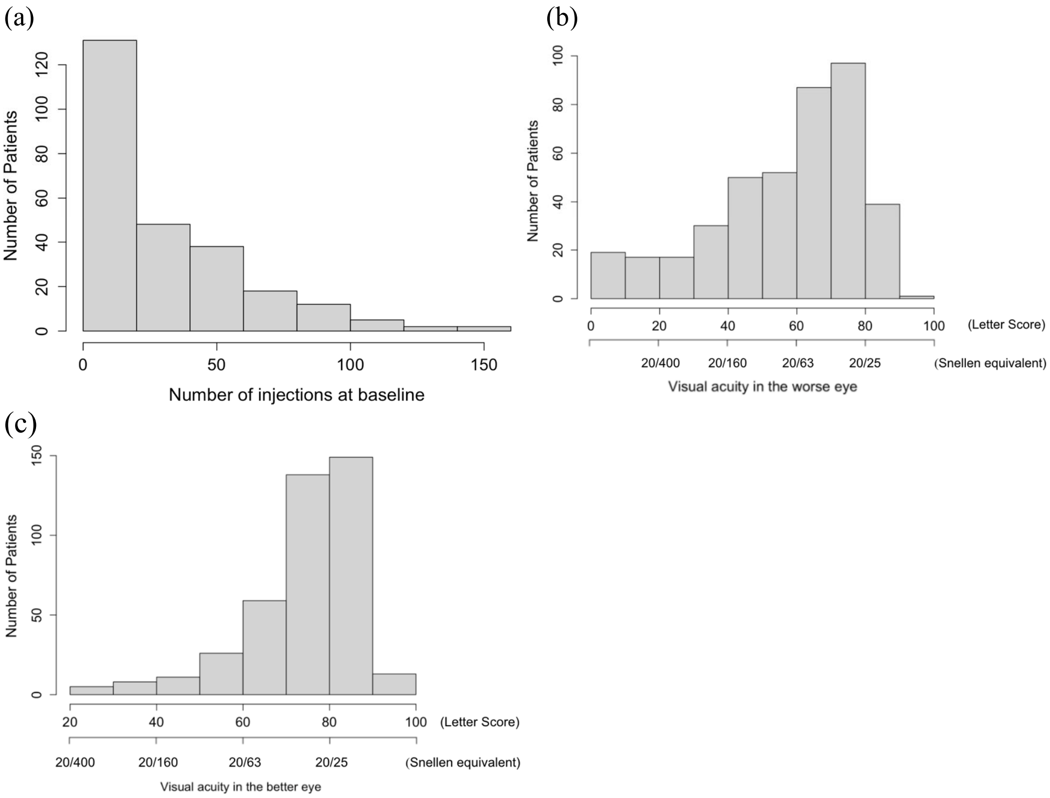

© 2021 Korot E et al. *JAMA Ophthalmology*.

eFigure 3. Age distribution of participants (a) and age distribution between female and male participants (b)

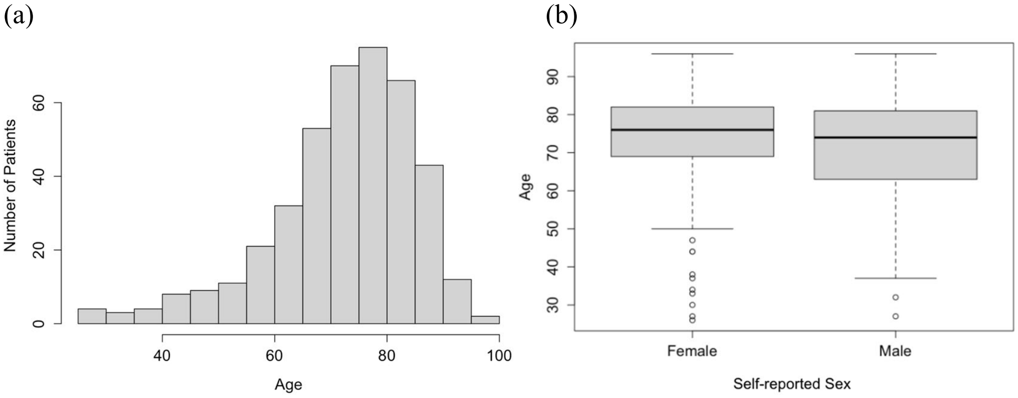

© 2021 Korot E et al. *JAMA Ophthalmology*.

eFigure 4. Change in SDH score and visual acuity

The HVM app, uses Shape Discrimination Hyperacuity (SDH) to detect metamorphopsia in the central degrees of vision as a metric of visual function. Figures show the change of SDH score by laterality (weeks) up to 17th week for active patients (a) and change of visual acuity over time for active patients (b), for both active and inactive patients (c). The 17th week represents the median number of weeks that active users continued to use the app.

(a)

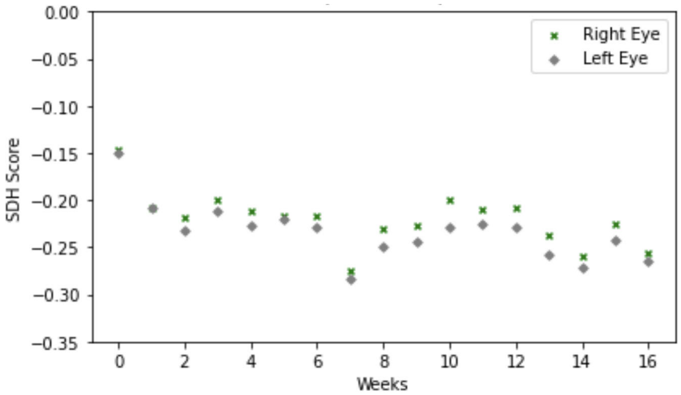

(b)

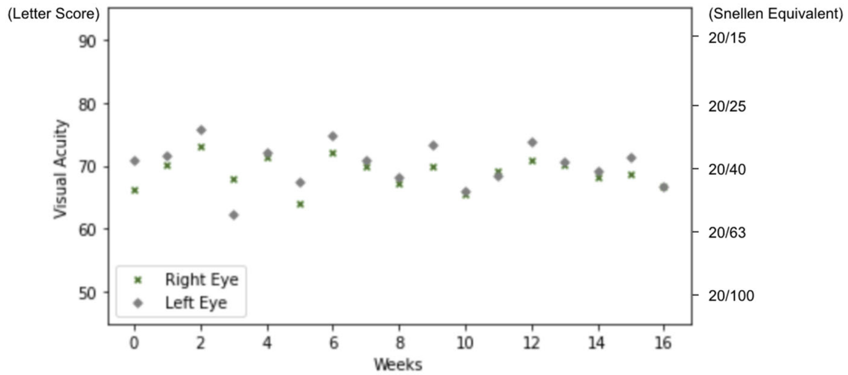

(c)

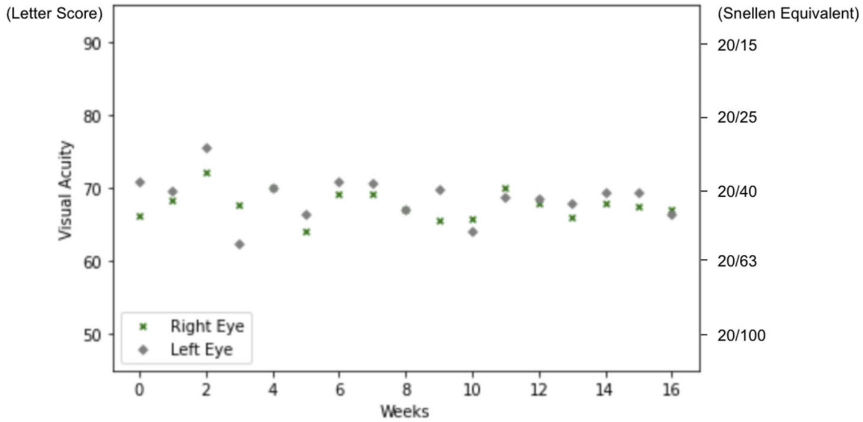

© 2021 Korot E et al. *JAMA Ophthalmology*.

eFigure 5. Histogram of use rate

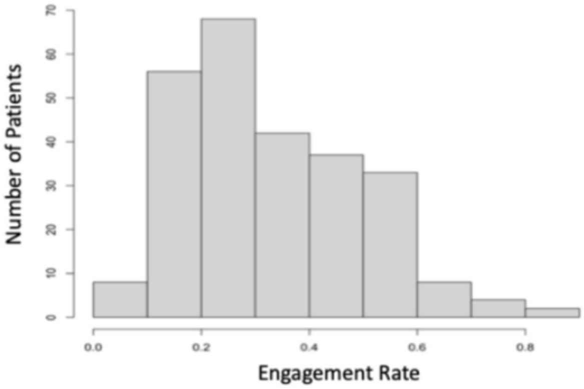

© 2021 Korot E et al. *JAMA Ophthalmology*.

eFigure 6. Correlation matrix of patient survey responses (Pearson correlation coefficients)

	Age	Q4	Q5	Q6	Q7	Q8	Q9	Q10	Q11	Q12	Q14	Q15	Q16	Q19	Q20	Q21	Q22	Q23	Q24	Q26
Age	1	-0.11	-0.14	-0.21	-0.13	-0.03	0.06	-0.39	-0.17	-0.04	0.26	-0.26	-0.12	-0.17	-0.02	-0.09	-0.1	-0.01	-0.08	0.18
Q4 Duration of use	-0.11	1	-0.04	0.02	-0.03	-0.01	-0.04	0.11	0.08	-0.07	-0.16	-0.03	-0.09	0.18	-0.24	0.09	0.09	-0.1	-0.07	0.11
Q5 How does the app make you feel?	-0.14	-0.04	1	0.39	0.58	0.3	-0.01	0.18	-0.07	-0.09	-0.07	0.01	0.04	0.31	-0.04	0	0.06	0.25	0.12	-0.12
Q6 Level of meeting expectations	-0.21	0.02	0.39	1	0.69	0.59	0.03	0.08	-0.11	-0.01	0.05	0.13	0.12	0.3	-0.03	-0.07	0.01	0.31	0.07	0.05
Q7 Level of satisfaction	-0.13	-0.03	0.58	0.69	1	0.62	-0.01	0.08	0	-0.05	0.07	0.09	0	0.37	-0.04	-0.11	0.14	0.43	0.17	-0.13
Q8 How likely to recommend?	-0.03	-0.01	0.3	0.59	0.62	1	0	-0.07	-0.08	0.02	0.08	0.05	-0.07	0.31	0.11	-0.02	0.04	0.49	0	-0.08
Q9 Is English your first language?	0.06	-0.04	-0.01	0.03	-0.01	0	1	0.23	0	0.09	-0.02	0.28	0.11	-0.04	-0.02	0.06	0.07	0.04	0.47	0.04
Q10 Level of comfort with modern technologies	-0.39	0.11	0.18	0.08	0.08	-0.07	0.23	1	0.34	0.1	-0.24	0.35	0.2	0.18	-0.07	0.09	0.15	-0.09	0.11	-0.02
Q11 Level of education	-0.17	0.08	-0.07	-0.11	0	-0.08	0	0.34	1	0.32	-0.15	0.09	0.02	0.01	-0.03	-0.01	0.02	0.03	0.09	-0.04
Q12 Level of income	-0.04	-0.07	-0.09	-0.01	-0.05	0.02	0.09	0.1	0.32	1	-0.05	0.12	-0.01	0	-0.02	0.02	0.03	-0.18	-0.18	0.03
Q14 Did you require assistance setting up the app?	0.26	-0.16	-0.07	0.05	0.07	0.08	-0.02	-0.24	-0.15	-0.05	1	0.08	-0.21	-0.16	0.04	0.04	0.03	0.12	-0.09	0.21
Q15 Do you use your own device?	-0.26	-0.03	0.01	0.13	0.09	0.05	0.28	0.35	0.09	0.12	0.08	1	0.15	0.15	-0.11	0.14	0.14	-0.03	0.15	0
Q16 Are you aware of the technical helpline?	-0.12	-0.09	0.04	0.12	0	-0.07	0.11	0.2	0.02	-0.01	-0.21	0.15	1	-0.04	0.22	0.16	-0.08	-0.15	-0.02	0.09
Q19 How easy was testing?	-0.17	0.18	0.31	0.3	0.37	0.31	-0.04	0.18	0.01	0	-0.16	0.15	-0.04	1	0.08	0.01	0.21	0.12	0.06	-0.12
Q20 Are you aware of in-app instructional videos?	-0.02	-0.24	-0.04	-0.03	-0.04	0.11	-0.02	-0.07	-0.03	-0.02	0.04	-0.11	0.22	0.08	1	0.03	-0.07	0.1	-0.13	0.07
Q21 Do you use the reminder feature?	-0.09	0.09	0	-0.07	-0.11	-0.02	0.06	0.09	-0.01	0.02	0.04	0.14	0.16	0.01	0.03	1	0.12	-0.04	-0.05	0.08
Q22 Do you want to receive your vision test results?	-0.1	0.09	0.06	0.01	0.14	0.04	0.07	0.15	0.02	0.03	0.03	0.14	-0.08	0.21	-0.07	0.12	1	-0.01	0.18	-0.38
Q23 Do you feel more reassured?	-0.01	-0.1	0.25	0.31	0.43	0.49	0.04	-0.09	0.03	-0.18	0.12	-0.03	-0.15	0.12	0.1	-0.04	-0.01	1	0.08	-0.14
Q24 Do you want to get more educational content?	-0.08	-0.07	0.12	0.07	0.17	0	0.47	0.11	0.09	-0.18	-0.09	0.15	-0.02	0.06	-0.13	-0.05	0.18	0.08	1	-0.28
Q26 Are you likely to continue using the app?	0.18	0.11	-0.12	0.05	-0.13	-0.08	0.04	-0.02	-0.04	0.03	0.21	0	0.09	-0.12	0.07	0.08	-0.38	-0.14	-0.28	1

© 2021 Korot E et al. *JAMA Ophthalmology*.

eFigure 7. Patient registration flow chart—Home Vision Monitoring

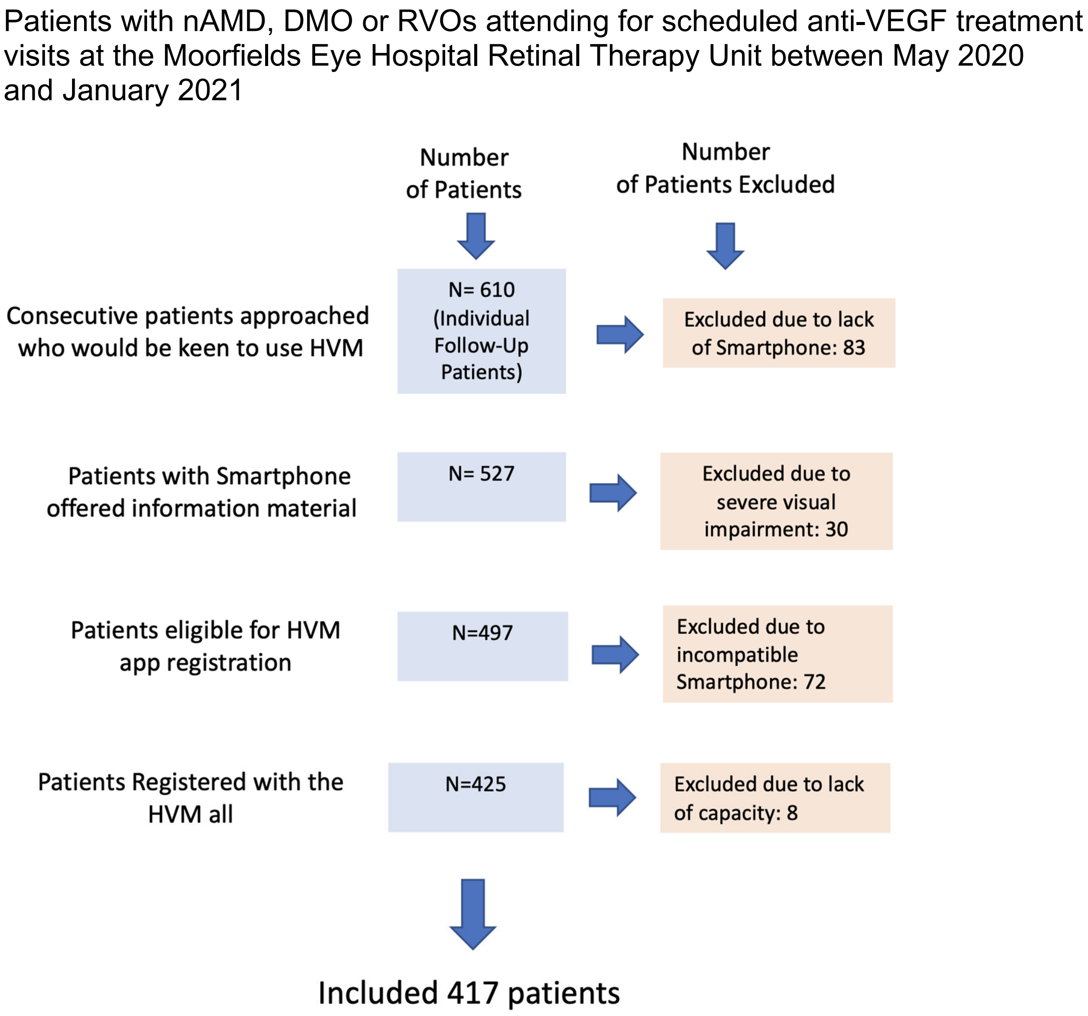

© 2021 Korot E et al. *JAMA Ophthalmology*.

eTable 1. Survey questions and responses

Section 1: General HVM Questions			
Question	Response	Number	Percent
Where did you hear about Home Vision Monitor (HVM)? (FT)	Moorfields Eye Hospital - Retinal Therapy Unit	102	87.2%
	MEH phone call	15	12.8%
Are you currently using HVM, and if so, what interested you in trying it? What were your expectations or reasons to use HVM? (FT)	Yes - to help with closer vision monitoring and benefit their eye health	65	55.6%
	Yes - to help research in eye disease	19	16.2%
	Yes - because doctor/hospital recommended it	24	20.5%
	Yes - no reason specified	5	4.3%
	No	4	3.4%
Q3: If you have not used HVM yet, could you kindly let us know why?(FT)	I need assistance with downloading HVM	0	0.0%
	I haven't downloaded HVM (haven't had a chance, etc.)	0	0.0%
	I did not receive a registration code	0	0.0%
	I have changed my mind about using HVM	0	0.0%
	I need further assistance with using HVM	0	0.0%
	Other (please Specify)	0	0.0%
	Felt vision too poor so patient and family decided there was no point in using the app	1	25.0%
	Too many things to take care of already regarding her vision and does not want to add more work for herself and the family	1	25.0%
	Feedback required from the test- would be worrying to not know the results of the test for the patient so has decided to discontinue use of hvm	2	50.0%
Q4: How long have you been using HVM? (FT)	2 months	11	9.4%
	3 months	38	32.5%

© 2021 Korot E et al. *JAMA Ophthalmology*.

	4 months	13	11.1%
	6 months	36	30.8%
	8 months	19	16.2%
Q5: How has using HVM to test your vision made you feel? (Likert)	Very bad	3	2.6%
	Bad	6	5.1%
	Neutral	48	41.0%
	Good	54	46.2%
	Very good	6	5.1%
Q6: On a scale of 1 to 5, 1 being the least and 5 being the most, how well have your expectations of HVM been met?	1	4	3.4%
	2	6	5.1%
	3	34	29.1%
	4	32	27.4%
	5	41	35.0%
Q7: On a scale of 1 to 5, 1 being the least satisfied and 5 being the most satisfied, how would you rate your overall HVM experience?	1	4	3.4%
	2	5	4.3%
	3	27	23.1%
	4	30	25.6%
	5	51	43.6%
Q8: On a scale of 1 to 5, 1 being the least likely and 5 being the most likely, how likely are you to recommend HVM to another person?	1	9	7.9%
	2	4	3.5%
	3	17	14.9%
	4	17	14.9%
	5	67	58.8%
Section 2: Patient Specific			
Question	Response	Number	Percent
Q9: Is English your first language? If not, which is your first language (FT)	YES	88	75.2%
	No (altogether)	29	24.8%
	No, Hebrew	1	0.8%
	No, Greek	1	0.8%

© 2021 Korot E et al. *JAMA Ophthalmology*.

	No, Swedish	1	0.8%
	No, Arabic	4	3.4%
	No, Urdu	3	2.6%
	No, Indian	1	0.8%
	No, Gujarati	3	2.6%
	No, Farsi	1	0.8%
	No, Spanish	2	1.7%
	No, Norwegian	1	0.8%
	No, Bengali	3	2.6%
	No, Philipino	3	2.6%
	No, French	1	0.8%
	No, Cantonese	2	1.7%
	No, Italian	1	0.8%
	No, Dutch	1	0.8%
Q10: How comfortable are you with using modern technologies such as smartphones/tablets and the internet?	1/ Very comfortable	31	27.4%
	2/comfortable	47	41.6%
	3/Neutral	12	10.6%
	4/ Not comfortable	23	20.4%
Q11: What is your highest level of education? (primary school, secondary school, college/university, post-graduate such as MSc or PhD)	Not answered	2	1.7%
	primary school	1	0.9%
	secondary school	37	31.6%
	higher education/ college	21	17.9%
	university	32	27.4%
	post gradute,MSc/PhD	24	20.5%
Q12: How would you self-describe your financial situation? (difficult, average, affluent)(MCQ)	average	77	65.8%
	affluent	27	23.1%
	average to affluent	3	2.6%

© 2021 Korot E et al. *JAMA Ophthalmology*.

	no answer	6	5.1%
	difficult	4	3.4%
Q13: Did you find any of the following a difficulty in using HVM (choose all that apply):	My phone is not very good/breaks down often	0	0.0%
	I'm not very comfortable using a Smartphone	4	6.9%
	I would prefer if the instructions were available in other languages (other than English)	5	8.6%
	I don't always have access to the internet through my phone (eg because I don't have wi-fi at home or I am often running out of credits)	0	0.0%
	I often need help from a relative/carer to use the app and one is not always available	4	6.9%
	No to all	45	77.6%
Section 3: HVM App Setup			
Question	Response	Number	Percent
From the following scale, how easy was it to set up HVM on your device?	Very Difficult	1	0.8%
	Difficult	1	0.8%
	Easy	38	31.4%
	Very Easy	81	66.9%
Q14: Have you received any help setting up HVM on your device? If so, at which of the following points did you find you would like assistance? (all that apply are selected)	Finding HVM in the App Store	83	29.5%
	Downloading HVM from the App Store	81	28.8%
	Registering yourself on the HVM app	83	29.5%
	No help needed	31	11.0%
	Other	3	1.1%
If you did receive assistance, please specify which of the following you received help from	Doctor	15	6.4%
	Other hospital worker	46	19.5%
	HVM customer support	2	0.8%
	Family member	32	13.6%
	No help needed	22	9.3%

© 2021 Korot E et al. *JAMA Ophthalmology*.

	Neighbour	2	0.8%
	Other (Please Specify)	0	0.0%
Q15: Is the application set up on your own personal device or a family member's?	Personal	96	40.7%
	husbands/wife/partner/family/ someone else	21	8.9%
Q16: Are you aware there is an HVM customer service team to assist you with any problems or questions you may have relating to HVM?	yes	55	45.5%
	no	65	53.7%
	no answer	1	0.8%
Q17: Anything else you would like to add regarding your experience with downloading and setting up HVM?	no	92	88.5%
	yes	12	11.5%
Section 4: HVM Vision Testing			
Question	Response	Number	Percent
Q18: How many times a week are you using HVM to monitor your vision?	2x	103	88.0%
	1x	10	8.5%
	3x	2	1.7%
	once every fortnight	2	1.7%
Q19: From the following scale, how easy is it to test your vision using HVM?	Very Difficult	1	0.9%
	Difficult	12	10.3%
	Easy	52	44.4%
	Very Easy	52	44.4%
Q14: Did you require any assistance in using HVM to monitor your vision? If so, which of the following individuals provided you with help?	Doctor	4	3.6%
	HVM customer support	0	0.0%
	Family member	15	13.4%
	none	93	83.0%
Q20: Are you aware that there are instructional videos on the application on how to test your vision?	yes	50	42.7%
	no	67	59.8%
Q21: Do you use the reminder feature in HVM, which sends you notifications when to test your vision? Why or why not? If yes,	no, didn't know about it	24	20.7%
	yes, it's good/ helpful	71	61.2%
	no, has a calendar on the phone	6	5.2%

© 2021 Korot E et al. *JAMA Ophthalmology*.

how helpful did you find this feature to assist with monitoring your vision? If no, would you be interested in showing you how to use this feature?	yes, but it reminds her on the wrong days - she would like help with resetting it	1	0.9%
	Yes but has had to silence it because of the grating voice of the lady! But still has visual prompts which she finds probably helpful	1	0.9%
	no and would like help with this feature	11	9.5%
	Yes, doesn't always do the test though	1	0.9%
	no, reminded by family	1	0.9%
Section 5: HVM Features and Improvements			
Question	Response	Number	Percent
Q22: Would you like to receive the results of your vision test from the HVM app? If yes, what form would you prefer to receive it in? (FT)	No, no results	13	11.1%
	Yes, As a monthly report	44	37.6%
	Yes, As an appendix to your clinical outcome letter after each injection appointment	20	17.1%
	Yes, Review it on the app each time you use it	40	34.2%
Q23: Does Home Vision Monitor offer you more reassurance that your disease is more closely checked by your care providers (doctor/optometrist/nurse)?	yes	96	82.1%
	no	21	17.9%
Please indicate which of the statements below you agree with more	I would like to receive the results of my vision test from HVM every time I use it even if my vision has not changed	53	45.3%
	I would like to receive the results of my vision test from HVM only when there is a significant change in my vision (either vision improved or worsened significantly)	27	23.1%
	I don't want to receive the results of my vision test from HVM as long as I know these are being reviewed by one of my care providers (doctor/optometrist/nurse) who will notify me if there is a significant change in my vision	37	31.6%

© 2021 Korot E et al. *JAMA Ophthalmology*.

Would you like to read or watch any educational content on HVM that is related to your diagnosis or treatment? For example, an article that talks about how a patient manages their injection treatment.	yes	60	51.3%
	no	57	48.7%
Q25: What would you like to see improved or changed on HVM? (FT)	A: No change needed	53	75.7%
	B: Finds it difficult to differentiate the shapes after 3 or 4 screens it becomes difficult. Quite a lot of guess work with the shapes	4	5.7%
	C: More feedback/results	10	14.3%
	D: A button that says I cannot see a difference	3	4.3%
Section 6: HVM Continued Engagement			
Question	Response	Number	Percentage
Q26: What would make you more likely to continue using the HVM app? (open-ended question)	receive test results	7	6.7%
	receive feedback when visiting the hospital	5	4.8%
	be notified if there is improvement in vision	4	3.8%
	more information on the app and more information provided when speaking with the recruiter	1	1.0%
	Knowing it made a difference to somebody/ helped people	11	10.5%
	nothing specific, happy to carry on as long as needed	71	67.6%
	Change the voice on the app.	1	1.0%
	Every so often if someone rings up and offers time for reflection	1	1.0%
	If they sort out the shapes all looking the same	1	1.0%
	Prefers to use with ongoing treatment. May not use it once she is discharged from the clinic and is told that her eyes are stable	1	1.0%

© 2021 Korot E et al. *JAMA Ophthalmology*.

	Will continue but will needs some kind of results to feel motivated to continue testing	1	1.0%
	Improved graphics	1	1.0%
Which of the following would help you continue to use the HVM app? (choose all that apply and rank them in terms if importance)	More reminders from the hospital	16	10.5%
	More feedback about my vision result	97	63.4%
	A dedicated support helpline to help me when I need instructions to use the app (not applicable if the patient is already aware of the customer service team and helpline in place)	4	2.6%
	More opportunities to ask questions to my care providers (doctor/optometrist/nurse)	19	12.4%
	More opportunities to share my experience/questions with other patients	8	5.2%
	no changes needed for them to continue	9	5.9%

© 2021 Korot E et al. *JAMA Ophthalmology*.

eTable 2. Type of univariable statistical tests performed depending on type of predictor and outcome variable

**Predictor / Outcome**	**Engaged or Active (binary)**	**Engagement Rate (continuous)**
**Categorical (binary)**	Fisher Test	t-test
**Categorical (more than 2 categories)**	Fisher Test	ANOVA
**Ordinal or Continuous**	Logistic Regression	Linear Regression

© 2021 Korot E et al. *JAMA Ophthalmology*.

eTable 3. Association of patient uptake with demographic and clinical predictor variables (active vs nonactive users)

						Multivariable Analysis
	**Active**	**Not Active**	**Odds Ratio [CI.95]**	**P-value**	**Estimate**	**P-value**
**Age**			0.982 [0.966;0.998]	.02	0.981[0.964;0.998]	.03
Median (IQR)	75.0 (66.0 - 80.0)	76.5 (68.0 - 84.0)				
Missing	3 (1.9%)	1 (0.4%)				
**Biological sex**			0.689 [0.449;1.054]	.07		
Female (baseline)	155 (60.1%)	81 (50.9%)				
Male	102 (39.5%)	75 (47.2%)				
**Ethnicity**			1.181 [0.776; .180]	.26		
British	112 (43.4%)	63 (39.6%)				
Not British	145 (56.2%)	93 (58.5%)				
**Diagnosis**			0.647 [0.404; 1.038]	.06		
AMD	188 (72.9%)	103 (64.8%)				
MRO	59 (22.9%)	50 (31.4%)				
**VA in worse eye (ETDRS)**			1.006 [0.997; 1.015]	.13		
Median (IQR)	65.0 (46.2 - 75.0)	59.0 (44.5 - 74.5)				
Missing	4 (1.6%)	4 (2.5%)				
**VA in better eye (ETDRS)**			1.017 [1.002; 1.033]	.01	1.012 [0.997;1.029]	.13
Median (IQR)	80.0 (72.2 - 85.0)	78.0 (67.5 - 83.5)				
Missing	4 (1.6%)	4 (2.5%)				
**Number of injections**			1.008 [1.000; 1.016]	0.02	1.045 [1.002;1.092]	0.04
Median (IQR)	20.0 (9.0 - 45.2)	16.0 (5.0 - 39.0)				
Missing	2 (0.8%)	2 (1.3%)				

© 2021 Korot E et al. *JAMA Ophthalmology*.

eTable 4. Associations of patient engagement rate with demographic, clinical, and survey predictor variables

			Univariable		Multivariable	
	**Engagement-rate < 0.3**	**Engagement-rate > 0.3**	**Effect [CI.95]**	**P-value**	**Effect [CI.95]**	**P-value**
**Age**	73 (62.5 - 79.0)	75 (68.25 - 81.00)	0.0014 [-0.0002; 0.003]	.08		
**Biological sex**			-0.0113 [-0.0527; 0.0302]	.59		
**Male**	54 (42.5%)	44(35.0%)				
**Female (baseline)**	73 (57.5%)	82(65.0%)				
**Ethnicity**			0.0280 [0.0128; 0.0688]	.18		
**British**	48 (37.5%)	63 (50%)				
**Non-British (baseline)**	80 (62.5%)	63 (50%)				
**Diagnosis**			0.0246 [-0.0209; 0.0701]	.29		
**nAMD**	90 (74.4%)	94 (77.0%)				
**MRO (baseline)**	31 (25.6%)	28 (23.0%)				
**VA in worse eye**	64 (46 - 74)	65(49 - 75)	0.0010 [0.0001; 0.0020]	.03	0.0001 [-0.0015; 0.0016]	.26
**VA in better eye**	80 (73 - 86)	80 (73 - 85)	0.0007 [-0.0009; 0.0023]	.36		
**Number of injections**	17.5 (9 - 37)	21.5 (10 - 56)	0.0008 [0.0001; 0.0014]	.03	0.00004 [-0.001; 0.0011]	.07
**Level of Comfort with Technology**			0.0241 [-0.0006; 0.0471]	.05	0.0314 [0.0069; 0.0551]	.02
**Level 1-2**	15 (28.3%)	4 (6.7%)				
**Level 3-5**	38 (71.7%)	56 (93.3%)				

© 2021 Korot E et al. *JAMA Ophthalmology*.

eTable 5. Univariable associations between patient compliance outcome and before-usage survey variables (compliant vs noncompliant users)

	Compliant	Non-Compliant	Odds Ratio [CI.95]	P-value
**English First Language**			1.329 [0.427;4.135]	.76
Yes	76 (76.0%)	12 (70.6%)		
No	22 (22.0%)	4 (23.5%)		
Missing	2 (2.0%)	1 (5.9%)		
**Using Own Device**			1.026 [0.264;3.978]	.97
Yes	80 (80.0%)	13 (76.5%)		
No	18 (18%)	3 (17.6%)		
Missing	2 (2.0%)	1 (5.9%)		
**Aware of Customer Service**			1.796 [0.615;5.243]	.43
Yes	48 (48.0%)	6 (35.3%)		
No	49 (49.0%)	10 (58.8%)		
Missing	3 (3.0%)	1 (5.9%)		
**Aware of Instructional Videos**			0.756 [0.269;2.130]	.79
Yes	39 (39.0%)	7 (41.2%)		
No	58 (58.0%)	9 (52.9%)		
Missing	3 (3.0%)	1 (5.9%)		
**Use Reminder Features**			1.058 [0.359;3.114]	.78
Yes	64 (64.0%)	10 (58.8%)		
No	33 (33.0%)	6 (35.3%)		
Missing	3 (3.0%)	1 (5.9%)		
**Wish to Receive Results**			0.147 [0.019;1.157]	.01
Yes	68 (68.0%)	16 (94.1%)		
No	29 (29.0%)	0 (0.0%)		
Missing	3 (3.0%)	1 (5.9%)		
**Sense of Reassurance**			1.912 [0.595;6.146]	.31
Yes	78 (78.0%)	11 (64.7%)		
No	17 (17.0%)	5 (29.4%)		
Missing	5 (5.0%)	1 (5.9%)		
**Watch Educational Content**			2.008 [0.676;5.960]	.28
Yes	53 (53.0%)	6 (35.3%)		
No	44 (44.0%)	10 (58.8%)		
Missing	3 (3.0%)	1 (5.9%)		
**Willing to Continue**			1.909 [0.402;9.069]	.52
Yes	21 (21.0%)	2 (11.8%)		
No	77 (77.0%)	14 (82.4%)		
Missing	2 (2.0%)	1 (5.9%)		
**Level of General Feeling**			4.252 [0.357;50.611]	.51
Median (IQR)	3.0 (3.0 - 4.0)	3.0 (3.0 - 4.0)		
Missing	1 (5.9%)	2 (2.0%)		

© 2021 Korot E et al. *JAMA Ophthalmology*.

**Level of Education**			1.030 [0.175;6.069]	.18
Median (IQR)	3.0 (3.0 - 4.0)	3.0 (3.0 - 4.0)		
Missing	1 (5.9%)	4 (4.0%)		
**Level of Financial**			0.919 [0.114;7.432]	.89
Median (IQR)	3.0 (3.0 - 5.0)	3.0 (3.0 - 5.0)		
Missing	1 (5.9%)	8 (8.0%)		

© 2021 Korot E et al. *JAMA Ophthalmology*.

eTable 6. Univariable associations between patient compliance outcome and after-usage survey variables (compliant vs noncompliant users)

	Compliant	Non-Compliant	Odds Ratio [CI.95]	P-value
**Level of HVM Expectation**			2.247 [0.301;16.762]	.62
Median (IQR)	4.0 (3.0 – 5.0)	4.0 (3.0 - 5.0)		
Missing	14 (14.0%)	4 (23.5%)		
**Level of Satisfaction**			4.733 [0.828;27.063]	.10
Yes	4.0 (3.0 - 5.0)	3.5 (3.0 - 4.2)		
Missing	2 (2.0%)	1 (5.9%)		
**Level of Recommendation to others**			2.416 [0.578;10.094]	.20
Median (IQR)	5.0 (3.0 - 5.0)	4.0 (2.5 - 5.0)		
Missing	7 (7.0%)	2 (11.8%)		
**Easy of Use**			1.539 [0.161;14.704]	.71
Median (IQR)	4.0 (4.0 - 5.0)	4.0 (3.8 - 5.0)		
Missing	3 (3.0%)	1 (5.9%)		
**Level of Comfortable with Technology**			0.701 [0.112;4.403]	.30
Median (IQR)	4.0 (3.0 - 5.0)	4.5 (3.5 - 5.0)		
Missing	3 (3.0%)	1 (5.9%)		

© 2021 Korot E et al. *JAMA Ophthalmology*.
